# Study of blend composition of nano silica under the influence of neutron flux

**DOI:** 10.1186/s40580-014-0021-7

**Published:** 2014-08-02

**Authors:** Elchin Huseynov, Adil Garibov, Ravan Mehdiyeva

**Affiliations:** Institute of Radiation Problems of Azerbaijan National Academy of Sciences, AZ 1143, B. Vahabzadeh 9, Baku, Azerbaijan

**Keywords:** Nanopowder, Nano silica, Nanomaterial, Neutron activation analysis, Neutron irradiation, Radioactivity, Radioisotopes, 81.07.Wx, 61.46. + w, 61.80.Hg, 28.20.Fc, 92.20.Td

## Abstract

Nano SiO_2_ compound with 160 m^2^/g specific surface area and 20 nm sizes has been irradiated continuously with neutron flux up to 20 hours in various periods in TRIGA Mark II type research reactor. The initial activities of different type radionuclides defined in the result of eight day activity analysis changes between wide range of 1,5 kBq- 1,5GBq. In the result of activity analysis carried out after the irradiation, the element content of 0,5% mixture existing in nano SiO_2_ compound has been defined with radionuclides of relevant element. It has been defined percentage amounts of elements in blend composition according to the performed activities.

## 1 Introduction

Silicon and its oxide compositions are widely applied in different fields of science and technology for their unique physical, physical-chemical properties and radiation- durability [1-9]. In real application conditions the surface of silicon-based materials and devices are covered with oxide layer. The influence of the oxide layer formed on surface on physical properties of base material and effective thickness in terms of defence from subsequent oxidation process are usually in nanometer form. On the other hand nano-size silicon and SiO_2_ are of great importance for their physical and surface physical-chemical properties.

Oxide compositions of silicon are widely used in electronics, radiation technology and radiochemical processes [1,2,5,9,10]. Conversion of ionizing radiation energy and transfer to surface levels is of great importance for radiative study of materials and radiation technology. From this point of view nano-size SiO_2_ is a perspective and at the same time an actual model system for study of radiation defect-formation, conversion and transfer processes of ionizing radiation energy. In recent years formation of nano size SiO_2_ compound and improvement of their purity degree are in the focus of attention of researchers [11-13]. At modern period the purity degree of nano silica has been brought to 99,5 – 99,9% and intensive scientific studies are being carried out in order to increase its value.

In the presented work under the influence of neutrons with 2×10^13^ n/cm^2^ s intensity it has been studied the dependence of activities of radioactive nucleus, formed in the result of neutron flux in powder and extruded shaped, 99,5% purity, 20 nm size nano SiO_2_ compound, on integral dose and decomposition time after irradiation. On the base of the achieved results it has been carried out the quality and quantity identification of blends in the composition of nano SiO_2_ compound.

The sample used in the presented work is widespread in the nature and met in crystalline forms as quartz, rock crystal, flint, opal, etc. In these crystals silica exists with different percentages and “Obsidian” natural glass has more SiO_2_ percentage (about 70-75% of SiO_2_) among natural crystals existing in the nature [14,15]. Nowadays, macro silica is treated with several methods and it was available to obtain maximum macro SiO_2_ (In Egypt) with 99,85% purity. However, the synthesis method of high purity SiO_2_ nanopowder in nanosize differs slightly and in this case, practically obtain of SiO_2_ nanopowder with perfect purity (100% purity) is almost impossible [16]. Thus, if purity of nano SiO_2_ powder obtained before was 75%, at present development of modern technology allowed obtaining SiO_2_ nanopowder with 99,9% purity [11-13]. Nowadays, nano SiO_2_ compounds with 99–99,9% purity are considered to be high purity nano SiO_2_ compounds and samples with this purity are of wide application. As it was mentioned before, nano SiO_2_ compound with 99,5% purity has been used during the experiment, so it can be considered as a high purity nano SiO_2_ compound. However, the sample contains 0,5% of impurities and even if at first sight it can seem a small indicator, it is very large value in nano-scale and molecule compiling. Therefore, if to take into account that there is approximately 10^22^ units of particles in atomic level in 1 g of SiO_2_ nanopowder, then 0,5% to be great value (approximately 5×10^19^ mixture particle) is obvious. Naturally, 0,5% mixture doesn’t impact the physical parameters of sample, but it clearly manifests itself during irradiation in the reactor and is of great significance.

## 2 Methods

From previous studies it is known that the specific surface area of nanomaterial used in the experiment is 160 m^2^/g, dimensions are 20 nm and some parameters of the used sample has been studied [17-19]. In the presented work the samples have been irradiated by neutron flux 2×10^13^ n/cm^2^s in central channel (Channel A1) of TRIGA Mark II light water pool type research reactor at full power (250 kW) in “Reactor Centre” of Jozef Stefan Institute (JSI) in Ljubljana city of Slovenia. It is important to note that the JSI TRIGA reactor has been thoroughly characterized [20-25] and the computational model used for computational characterization has been thoroughly verified and validated [26,27] against several experiments. It should be mentioned that in this channel the parameters of neutron flux at full power mode are 5.107×10^12^ cm^−2^ s^−1^ (1 ± 0.0008, E_n_ < 625 eV) for thermal neutrons, 6.502x10^12^ cm^−2^ s^−1^ (1 ± 0.0008, E_n_ ~ 625 eV ÷ 0.1 MeV) for epithermal neutrons, 7.585x10^12^ cm^−2^ s^−1^ (1 ± 0.0007, E_n_ > 0.1 MeV) for fast neutrons and finally for all neutrons the flux density in central channel is 1.920×10^13^ cm^−2^ s^−1^ (1 ± 0.0005) [20,26].

**Figure 1:**
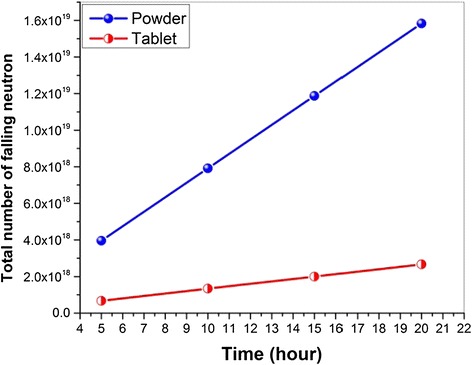
**Dependence of neutrons falling on the sample on irradiation period.**

The powdered nano SiO_2_ has been irradiated in a special cylindrical aluminium container. Density of powdered nano SiO_2_ compound is ρ_powder_ = 0,1 g/cm^3^ (density in packaging is approximately ~0,3 g/cm^3^), volume of the sample in cylinder-shaped radiation packaging is V_powder_ ≈ 2,3 cm^3^, the area of the sample which is exposed to neutron flux is S_powder_ ≈ 10 cm^2^. The sample which is approximately same amount ~0,7 g being pressed in a special press machine has been put in a special form and its parameters are: ρ_tablet_ = 2,9 g/cm^3^, V_tablet_ ≈ 0,2 cm^3^, S_tablet_ ≈ 1,8 cm^2^. The prepared experiment sample was irradiated firstly for 5 minutes evaluate the final activity after 20 hour radiation. Then other 8 samples (each one apx. 0,7 g) were divided into 4 groups and each of them irradiated continuously for 5, 10, 15, 20 hours by neutron 2×10^13^ cm^−2^ s^−1^ in central channel (Channel A1) of TRIGA Mark II type research reactor at full power (250 kW). Both forms of the samples (4 powder samples and 4 tablets) have been irradiated in 2×10^13^ n/cm^2^s intensity of neutron flux. Absorption dose values of the studied samples have been defined according to geometric dimensions of powdered and tablet-formed samples, intensity of irradiation, irradiation periods, density of influencing neutron flux and energy spectra of neutrons. The value of neutron flux for powdered samples is 3,95×10^18^ ÷ 1,58×10^19^ and for tablet-formed ones it changes within the neutron range 6,67×10^17^ ÷ 2,67×10^18^. The dependence of neutrons falling on the sample on irradiation period for samples of both types has been given in Figure 1. As it is seen, the interacting neutron flux and thus the amount of absorbed neutrons in powdered sample is approximately 25 times more than in tablet-formed.

Radionuclides being formed in composition of nano SiO_2_ compound after the interaction with neutron have been analysed in spectrometers “Ortec HPGe detectors (Coaxial, Low and Well-Type)” and “Canberra coaxial HPGe detector”. The radioactivity, isotope composition and amount of blend elements of the irradiated samples have been determined on technique [28-31].

## 3 Results and discussions

**Figure 2:**
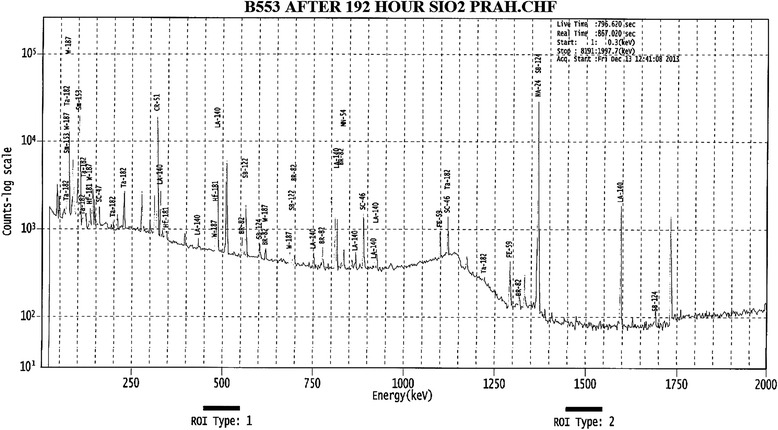
**Gamma spectrum of nano SiO**_**2**_ **sample, irradiated by neutrons with 2x10** ^**13**^ **n/cm** ^**2**^ **s flux for τ = 5,10,15,20 hours, in 192 hours after irradiation.**

The main probable process of interaction of neutrons with substance is radiation capacity [28]:

1 $$ n+{}^A Z\to {}^{A+1} Z^{*}\to {}^{A+1} Z+\gamma $$

here ^A^Z – irradiated isotope, ^A+1^Z^*^ - excited nucleus formed as a result of neutron capacity and γ - second gamma rays. Radioactive excited samples are formed in the result of the processes shown in irradiated samples. Their identification has been studied with the method of gamma spectroscopy. γ – ray intensities appropriate to nuclear transitions in gamma spectra are different depending on irradiation period and decay constants. One of these spectra has been given in Figure 2 as an example.

After neutron irradiation it has been studied the change of samples’ activities after 192 hours. It has been determined that initial radioactivities of irradiated samples change between the ranges 1,5 kBq – 1,5GBq (powder and tablet each one total four sample approximate 3 g). Define of concentrations of elements is conducted on the base of the activities originated in appropriate energy range. The activity is determined on the following formula based on nuclear constants [28]:

2 $$ \begin{array}{l} A=\sigma \varPhi \left(\frac{m}{M}\right){N}_A\varTheta {P}_{\gamma}\xi \left(1- \exp \left(-\lambda {\tau}_{ray}\right)\right)\times \\ {}\left(1- \exp \left(-\lambda {\tau}_{m es}\right)\right) \exp \left(-\lambda {\tau}_{cool}\right)\end{array} $$

here, A – measured activity (Bq), σ – cross section of activation of defined isotopes (cm^2^), Φ – neutron flux (n/cm^2^s), m – weight of defined element (g), M – atomic weight of defined element (g/mol), N_A_ – Avogadro number (1/mol), Θ – distribution of activated isotope, P_γ_ –distribution probability of E energy gamma quantum, ξ – defect efficiency according to E energy, λ – decay constants of the formed isotopes, τ_ray_, τ_mes_, τ_cool_– irradiation, measurement and cooling time interval of samples correspondingly.

**Figure 3:**
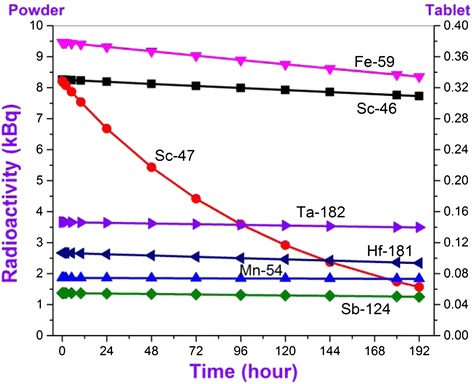
**Dependence of activities of conditional I group radionuclides being formed in nano SiO**_**2**_ **under the influence of neutron flux on measurement time.**

**Figure 4:**
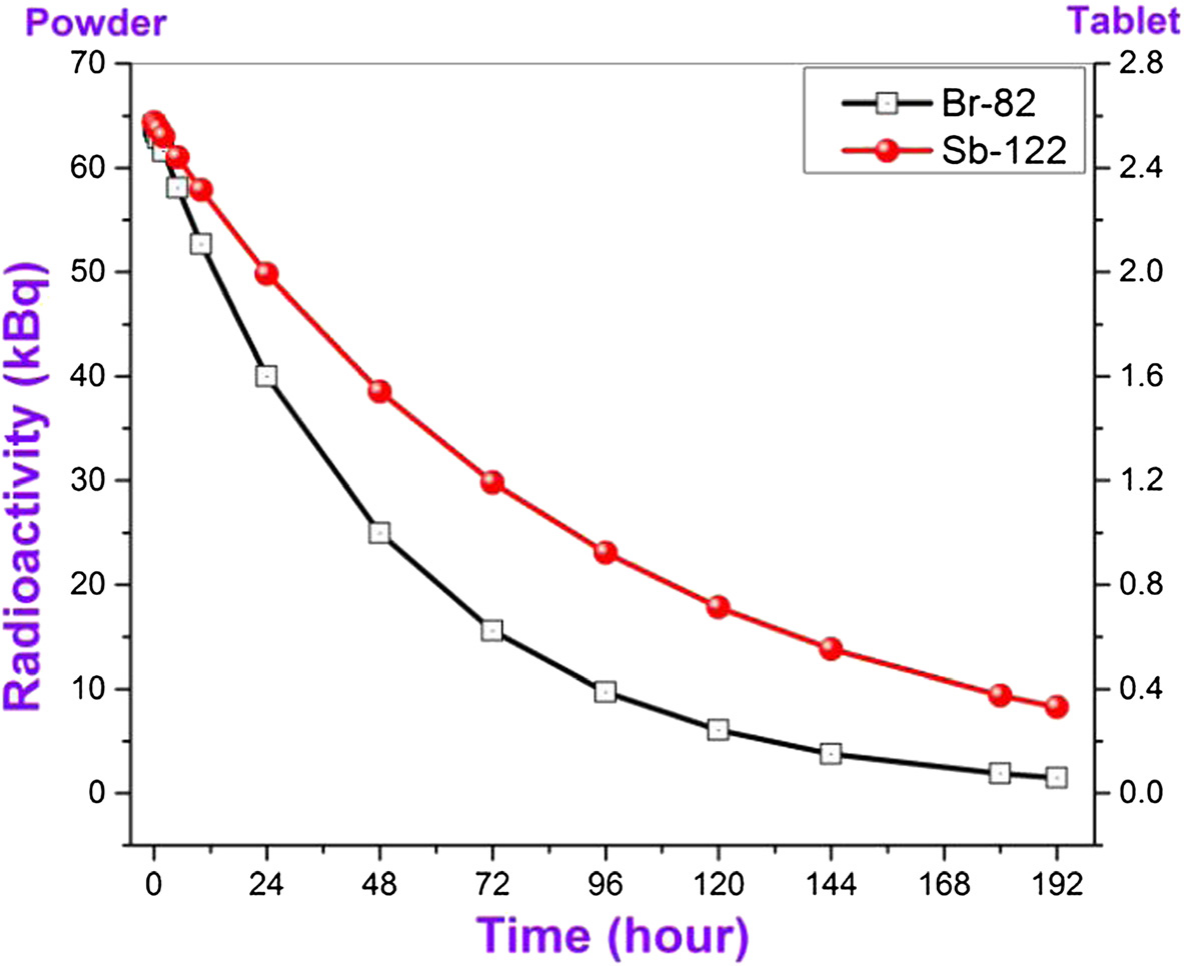
**Dependence of activities of conditional II group radionuclides being formed in nano SiO**_**2**_ **under the influence of neutron flux on measurement time.**

**Figure 5:**
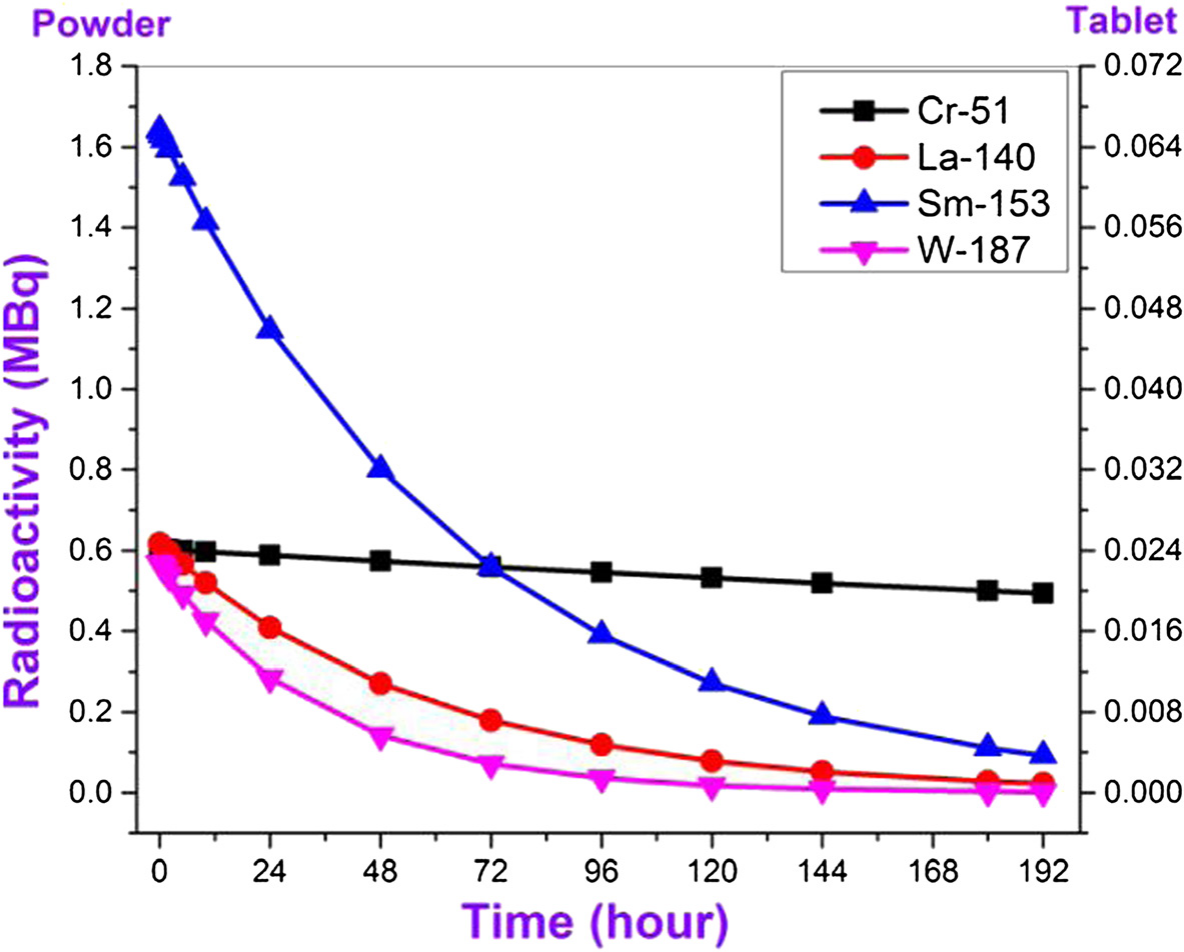
**Dependence of activities of conditional III group radionuclides being formed in nano SiO**_**2**_ **under the influence of neutron flux on measurement time.**

**Figure 6:**
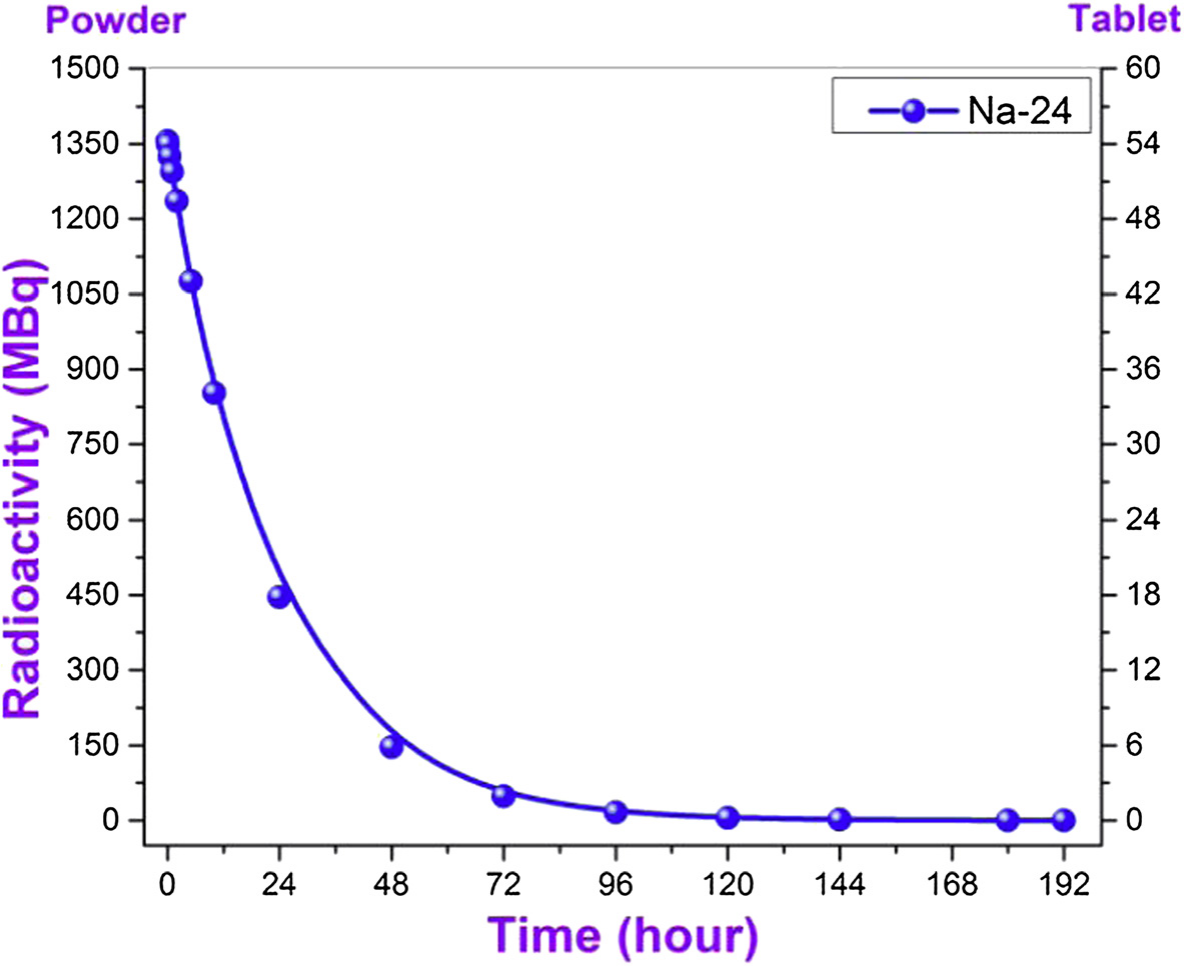
**Dependence of activity of** ^**24**^ **Na isotope included to the conventional IV group originated in nano SiO**_**2**_ **under the influence of neutron flux on measurement time.**

Activities of newly formed radioactive isotopes observed in irradiated samples change correspondingly to decay constants. The initial activities of different type radionuclides defined in the result of eight day activity analysis changes between wide range of 1,5 kBq- 1,5 GBq. Just for this reason we decided to divide these elements into four groups. The observed radioactive isotopes can be divided conditionally into four groups as I – A ≤ 10 kBq, II – A ≤ 100 kBq, III – A ≤ 2 MBq and IV – A ≤ 1,5 GBq for their activities. Observation time dependence of initial activities of the observed radioactive isotopes on conventional groups has been given in figures. Dependency of radioactivity on observation time has been defined for both powdered and tablet-formed nano SiO_2_ (Figures 3, 4, 5 and 6). It should be noted that all used graphics cover an eight day- period and elapsed time was denominated in hours. First, let’s review radionuclides with activity up to 10 kBq which is generated in nanocompound with the influence of neutron flux (Figure 3).

In general, it has been observed 7 types of radionuclides, thus their half-life changes from 80,4 hours up to 748,2 hours. For living periods, the observed “I group” radionuclides can be lined up as Sc-47 (80,4 hour), Hf-181 (1017,36 hour), Fe-59 (1067,88 hour), Sb-124 (1444,8 hour), Sc-46 (2010,96 hour), Ta-182 (2753,76 hour) and Mn-54 (7489,2 hour).

The activity of radionuclides included inside II group is up to 70 kBq (Figure 4). It has been observed two type Br-82 and Sb-122 radionuclides that their half-life are 35, 3 h for Br-82 and 64, 8 h for Sb-122 respectively. As it is seen from Figure 4, after 8 days the activity of Br-82 isotope decreased approximately down to 5 kBq, and the activity of Sb-122 down to 10 kBq.

The activity of only one of four radionuclides including to the third group is approximately up to 2 Mbq (for comparison: the activity in tablet form of relevant radionuclide is approximately up to 72 kBq, right column in the fig.) and the activity of other three radionuclides do not exceed 0,6 MBq (Figure 5). Half-lifes of observed radionuclides are 24 h (W-187), 40,32 h (La-140), 46,28 h (Sm-153) and 665 h (Cr-51) and after eight days activity for Cr-51 decreased up to approximately 0,5 MBq, and 0,1 MBq for other ones.

Only one element is included to the last IV group that its activity was approximately up to 1,5 GBq (for comparison: the activity in tablet form of relevant radionuclide is approximately 54 MBq, right column in the fig.). The half-life of observed high activity Na-24 isotope is 14,95 hours and at the end of measurement day the activity of Na-24 isotope decreased approximately down to 0,2 MBq (Figure 6). It can be said that the major part of mixture containing Na-24 isotope which is more active than other ones is the element of Na.

It is seen from the dependency given in Figure 4 that activities of isotopes with small radioactive decay constant and which are relatively high within half decay, change little during experimental observation and it is expected the influence of their radioactive decay during subsequent study of nano SiO_2_ compound properties. According to comparison between activities of isotopes defined experimentally and activities of standards taken in the same condition and weight, it has been defined the amount of each element in the content of nano SiO_2_ compound [28]:

3 $$ {C}_{sam}={C}_{st}\frac{A_{sam}}{A_{st}} $$

here, C_sam_ – concentration of the shown element isotope in nano SiO_2_, C_st_ – concentration of comparison standard in the same isotope, A_sam_ and A_st_ are activities of sample and standard, respectively. If weights of sample and standards are different, the concentration of the element [28-31] is defined on the given technique. The obtained results have been given in Table 1.

**Table 1:** **Isotopes generated by neutron activation in nano SiO**_**2**_ **, number of neutrons spent on activation of one isotope and number of isotopes in percent in sample**

**Stable isotope**	**Radioisotope**	**Number of capture neutron in each nucleus**	**Apx. amount of blend (%)**
Na-23	Na-24	+1	~0,3%
La-139	La-140	+1	~0,1%
Cr-50(52)	Cr-51	+1	
Sm-150(152)	Sm-153	+3	
W-184(186)	W-187	+3	
Sb-121	Sb-122	+1	<0,1%
Sb-123	Sb-124	+1	
Br-81	Br-82	+1	
Fe-56(57,58)	Fe-59	+3	<0,1%
Sc-45	Sc-46	+1	
Sc-45	Sc-47	+2	
Ta-181	Ta-182	+1	
Hf-178(179,180)	Hf-181	+3	
Fe-54(n,p)	Mn-54	+1	

In general, it has been given in tables the different terms’ radioactivity of radionuclides generated in 0,5% mixture during the influence of neutron flux to 99,5% purity nano SiO_2_, their initial isotopes and approximate amount. In Table 2 III and IV group radioisotopes and their activities have been shown with MBq and lined up in the form of initial activity increase.

**Table 2:** **Conditional III and IV radioisotope groups generated in nano-compound under the influence of neutron flux**

**Time (hour)**	**Radioactivity (MBq)**				
	**Na-24**	**Sm-153**	**La-140**	**Cr-51**	**W-187**
0	~1355	~1.6	~0.62	~0.604	~0.568
0.1	~1350	~1.6	~0.61	~0.603	~0.566
0.5	~1325	~1.6	~0.61	~0.603	~0.56
1	~1295	~1.6	~0.60	~0.602	~0.55
2	~1235	~1.59	~0.59	~0.601	~0.53
5	~1075	~1.52	~0.57	~0.6	~0.49
10	~850	~1.41	~0.52	~0.59	~0.42
24	~445	~1.14	~0.41	~0.58	~0.28
48	~146	~0.8	~0.27	~0.57	~0.14
72	~48	~0.55	~0.18	~0.56	~0.07
96	~15	~0.38	~0.12	~0.55	~0.04
120	~5	~0.27	~0.08	~0.53	~0.02
144	~1.7	~0.19	~0.05	~0.52	~0.01
180	~0.3	~0.11	~0.03	~0.5	~0.003
192	~0.2	~0.09	~0.02	~0.49	~0.002

In Table 3 I and II group radioisotopes and their activities have been shown with kBq and lined up in the form of initial activity increase. It should be mentioned that radioisotopes of the elements Si and O which are the main part of the sample, which may arise, have a very small existence period and in our discussions they have not been considered. So, Si-31 has short decay-time (~2.5 h) in 20 h irradiation, there could be produced some P which is beta emitter and cannot be detected by gamma detector. In general, in Table 1 it has been depicted initial isotopes and their amounts in percent according to blend radioisotopes generated under the influence of neutron flux.

**Table 3:** **Conditional I and II radioisotope groups generated in nano-compound under the influence of neutron flux**

**Time (hour)**	**Radioactivity (kBq)**								
	**Sb-122**	**Br-82**	**Fe-59**	**Sc-46**	**Sc-47**	**Ta-182**	**Hf-181**	**Mn-54**	**Sb-124**
0	~64.4	~64	~9.46	~8.25	~8.21	~3.67	~2.67	~1.86	~1.37
0.1	~64.3	~63.9	~9.46	~8.25	~8.2	~3.67	~2.67	~1.86	~1.37
0.5	~64.0	~63.44	~9.46	~8.25	~8.17	~3.66	~2.67	~1.86	~1.37
1	~63.7	~62.8	~9.45	~8.25	~8.14	~3.66	~2.66	~1.86	~1.37
2	~63	~61.6	~9.45	~8.24	~8	~3.65	~2.66	~1.86	~1.37
5	~61	~58	~9.43	~8.24	~7.87	~3.65	~2.65	~1.85	~1.36
10	~58	~53	~9.4	~8.23	~7.53	~3.65	~2.64	~1.85	~1.36
24	~50	~40	~9.32	~8.19	~6.67	~3.64	~2.62	~1.85	~1.35
48	~39	~25	~9.17	~8.12	~5.43	~3.62	~2.58	~1.85	~1.34
72	~30	~15	~9	~8.05	~4.41	~3.6	~2.54	~1.84	~1.32
96	~23	~10	~8.8	~7.98	~3.6	~3.57	~2.5	~1.84	~1.31
120	~18	~6	~8.7	~7.92	~2.9	~3.55	~2.45	~1.84	~1.29
144	~14	~4	~8.6	~7.85	~2.4	~3.53	~2.4	~1.83	~1.28
180	~9	~2	~8.4	~7.75	~1.7	~3.5	~2.35	~1.82	~1.25
192	~8	~1.5	~8.3	~7.72	~1.6	~3.48	~2.34	~1.82	~1.25

## 4 Conclusions

It has been carried out identification of radioactivity appeared in nano SiO_2_ under the influence of neutron flux and isotopes that formed radioactivity. It has been revealed dependency of samples’ activity and dose amount on irradiation time and sample dispersity. It has been defined that powdered nano SiO_2_ possess an activity approximately 25 times higher than the samples made as a tablet in special press form due to the interaction field with neutron to be big. Dependencies of radio activities of the revealed isotopes on observation time and amount of blend elements in percent have been defined. In the studied nano SiO_2_ samples it has been revealed the isotopes possessing relatively large half-decay time and these isotopes are suggested to be considered in explanation of physical properties of nano SiO_2_ compound within the period after irradiation.
